# Advances in Computational Methods for Genetic Diseases

**DOI:** 10.1155/2015/645649

**Published:** 2015-06-18

**Authors:** Francesco Camastra, Roberto Amato, Maria Donata Di Taranto, Antonino Staiano

**Affiliations:** ^1^Department of Science and Technology, University of Naples Parthenope, Centro Direzionale, Isola C4, 80143 Napoli, Italy; ^2^Wellcome Trust Centre for Human Genetics, University of Oxford, Oxford OX3 7BN, UK; ^3^IRCCS SDN, Via E. Gianturco 113, 80143 Napoli, Italy

Genetic diseases are a wide group of diseases in which the etiopathogenesis is caused by or related to genetic factors. The role of genetics in the disease development can be more or less relevant depending on the specific characteristics of the disease, and a wide spectrum of complexity exists.

Monogenic diseases, for example, are directly caused by defects in a specific gene whereas complex and polygenic diseases are generally caused by the interactions between multiple genes or between genetic and environmental factors. To the last category belong many forms of cancer, an uncontrolled growth of cells with alterations of the genetic materials.

In the last decade, a large amount of experimental data has become available, so the identification of strategies to process and, most importantly, interpret them is crucial. The massive volume of data, both in terms of quantity and of dimensionality, and their heterogeneity and low signal-to-noise ratio are just some of the most obvious challenges that they present. To give an example, single nucleotide DNA mutations are one of the most common factors analysed in relation to the development of a genetic disease. However, this sometimes translates into dealing with millions of variants measured across thousands of individuals, where only a handful are informative. In fact, other more complex factors, such as gene expression, could play a significant role.

The aim of this special issue is to review the recent advances in computational methods concerned with genetic diseases.

The issue received sixteen submissions; each one was referred by at least two international reviewers that we warmly thank for their time. Six papers have been accepted for the publication.

“A New Approach for Mining Order-Preserving Submatrices Based on all Common Subsequences” by Y. Xue et al. proposes, in the context of gene expression data, a pattern-based subspace clustering or OPSM (order-preserving submatrix model), based on frequent sequential pattern. The approach has been experimentally proven to be able to discover the biological significant OPSMs and deep OPSMs exhaustively.

“Evolutionary Influenced Interaction Pattern as Indicator for the Investigation of Natural Variants Causing Nephrogenic Diabetes Insipidus” by S. Grunert and D. Labudde is devoted to the application of a high-throughput analysis method based on motif conservation among proteins of the same protein family for analysis of interacting sequences. This investigation can help to analyze the pathogenic impact of mutations causing alterations in interacting regions of a protein. This analysis has been applied on membrane proteins, in particular to the aquaporin 2 whose mutants are involved in nephrogenic diabetes insipidus.

“Unified Modeling of Familial Mediterranean Fever and Cryopyrin Associated Periodic Syndromes” by Y. Bozkurt et al. describes a unifying dynamical model for Familial Mediterranean Fever (FMF) and Cryopyrin Associated Periodic Syndromes (CAPS) in the form of coupled nonlinear ordinary differential equations. The authors perform a comprehensive bifurcation analysis of the model and show that it exhibits three modes, capturing the healthy, FMF, and CAPS cases. They present extensive simulation results for the model that match clinical observations.

“Enhancing the Lasso Approach for Developing a Survival Prediction Model Based on Gene Expression Data” by S. Kaneko et al. presents a novel improvement to the lasso approach, one of the most widely used method to correlate gene expression data with cancer patients' survival. This new algorithm significantly increases the ability to identify “true positives” and its validity is shown on both simulated and real data.

“Statistical and Computational Methods for Genetic Diseases: An Overview” by Francesco Camastra, Maria Donata Di Taranto, and Antonino Staiano gives a survey of statistical and computational methods used to analyse the pathogenic role of sequence variants as well as to identify genetic markers of complex diseases by association studies, meta-analysis, and expression studies.

“Optimization and Corroboration of the Regulatory Pathway of p42.3 Protein in the Pathogenesis of Gastric Carcinoma” by Y. Hao et al. provides important research directions for exploring the mechanism of action of p42.3 protein in gastric cancer. Through a Bayesian network model, the potential important role of p42.3 is verified by both theoretical analysis and preliminary test.

We hope that the readers of this journal will find in the issue interesting papers and that this can encourage and foster further research on computational methods for genetic diseases.

*Francesco Camastra* *Francesco Camastra* *Roberto Amato* *Roberto Amato* *Maria Donata Di Taranto* *Maria Donata Di Taranto* *Antonino Staiano* *Antonino Staiano*

